# Outdoor Spaces, Transportation, and Environmental Justice: A Qualitative Interpretive Meta-Synthesis

**DOI:** 10.1093/geroni/igaa057.2463

**Published:** 2020-12-16

**Authors:** Noelle Fields, Kristen Ravi, Holly Dabelko-Schoeny

**Affiliations:** 1 The University of Texas at Arlington, Arlington, Texas, United States; 2 University of Texas at Arlington, Arlington, Texas, United States; 3 The Ohio State University, Columbus, Ohio, United States

## Abstract

Age-friendly environments aim to promote healthy and active aging by building and maintaining capacity across the life course and allow people who have a loss of capacity to continue engaging in activities that they value. Age-friendly community assessments are being conducted worldwide. This qualitative interpretive meta-synthesis (QIMS) aims to create a rich description of older adults’ experiences with outdoor spaces and buildings as well as transportation as part of an age-friendly assessment. The themes that emerged regarding older adults’ experiences with outdoor space and buildings included 1) accessibility and 2) appropriate infrastructure. Regarding transportation, the theme of accessibility included subthemes of 1) availability and 2) affordability. Further reduction indicated that age-friendliness can be conceptualized as environmental justice (EJ). The three areas of EJ including distributional justice, procedural justice, and recognition provide a helpful framework for social workers and their interdisciplinary partners to systematically document and evaluate their age-friendly community efforts.

